# Response of SII cortex to ipsilateral, contralateral and bilateral flutter stimulation in the cat

**DOI:** 10.1186/1471-2202-6-11

**Published:** 2005-02-14

**Authors:** Mark Tommerdahl, Stephen B Simons, Joannellyn S Chiu, Vinay Tannan, Oleg Favorov, Barry Whitsel

**Affiliations:** 1 Department of Biomedical Engineering, University of North Carolina, Chapel Hill, NC 27599, USA; 2 Department of Cellular and Molecular Physiology, University of North Carolina, Chapel Hill, NC 27599, USA

## Abstract

**Background:**

A distinctive property of SII is that it is the first cortical stage of the somatosensory projection pathway that integrates information arising from both sides of the body. However, there is very little known about how inputs across the body mid-line are processed within SII.

**Results:**

Optical intrinsic signal imaging was used to evaluate the response of primary somatosensory cortex (SI and SII in the same hemisphere) to 25 Hz sinusoidal vertical skin displacement stimulation ("skin flutter") applied contralaterally, ipsilaterally, and bilaterally to the central pads of the forepaws. A localized increase in absorbance in both SI and SII was evoked by both contralateral and bilateral flutter stimulation. Ipsilateral flutter stimulation evoked a localized increase in absorbance in SII, but not in SI. The SII region that responded with an increase in absorbance to ipsilateral stimulation was posterior to the region in which absorbance increased maximally in response to stimulation of the contralateral central pad. Additionally, in the posterior SII region that responded maximally to ipsilateral stimulation of the central pad, bilateral central pad stimulation approximated a linear summation of the SII responses to independent stimulation of the contralateral and ipsilateral central pads. Conversely, in anterior SII (the region that responded maximally to contralateral stimulation), bilateral stimulation was consistently less than the response evoked from the contralateral central pad.

**Conclusions:**

The results indicate that two regions located at neighboring, but distinctly different A-P levels of the anterior ectosylvian gyrus process input from opposite sides of the body midline in very different ways. The results suggest that the SII cortex, in the cat, can be subdivided into at least two functionally distinct regions and that these functionally distinct regions demonstrate a laterality preference within SII.

## Background

There is general agreement that in cats and monkeys (and presumably in humans) the spike discharge activity a mechanical stimulus sets up in rapidly adapting (RA), slowly adapting (SA), and Pacinian (PC) skin mechanoreceptors is projected centrally, at short latency and with relatively minor transformation, to primary somatosensory cortex (both SI and SII) in the contralateral hemisphere. There is no consensus, however, about the way in which the stimulus-evoked response in the ipsilateral hemisphere contributes to cerebral cortical somatosensory information processing and somatosensation. A distinctive property of SII is that it is the first cortical stage of the somatosensory projection pathway that integrates information arising from both sides of the body. Similar to SI, SII possesses a clear topographic organization [1], but unlike SI, a significant fraction of SII neurons possess bilateral receptive fields (RFs). The fraction of SII neurons with bilateral RFs varies from one topographic region of SII to the next. While ipsilateral input to SII is widely accepted, the role(s) of this input in somatosensory information processing remains uncertain. To investigate the effects on SII input deriving from mechanoreceptors in ipsilateral skin regions, the technique of optical intrinsic signal (OIS) imaging was used to assess the impact on SII of ipsilateral input on the response of SII to a contralateral input. The responses to contralateral, ipsilateral and bilateral vibrotactile stimulation (25 Hz – "flutter") of the forepaw of the cat were quantified and compared to make this assessment. Although evoked responses in both SI and SII were imaged in the studies, the primary focus of this report is the response of SII to the aforementioned stimuli.

## Results

**Figure 1:**
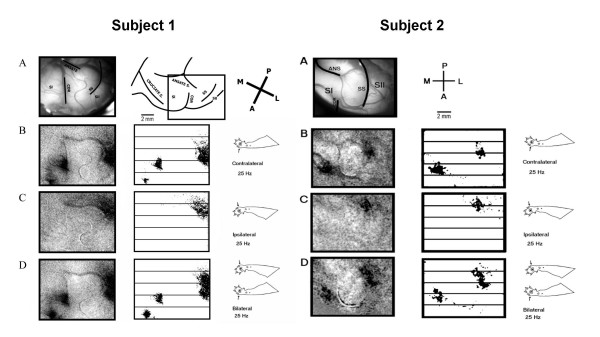
Cat SI and SII optical responses to 25 Hz vibrotactile stimulation of the forepaws (2 subjects). **A**. View of the cortical surface, showing the vascular pattern and coronal (COR), ansate (ANS), and suprasylvian (SS) sulci. Exposed portions of SI and SII are indicated. *Left hand column of Subject 1 and Subject 2 *: Averaged difference images for responses evoked by (**B**) contralateral, (**C**) ipsilateral and (**D**) bilateral stimuli. Adjacent thresholded images of responses evoked by the three modes of stimulation – horizontal grid lines facilitate comparison of the position of loci of the evoked responses and are spaced 2 mm apart. Stimulus sites are indicated by figurines. Note that in both subjects, ipsilateral stimulation evokes a response posterior to the response evoked by contralateral stimulation. Scale bar is 2 mm. Orientation of images indicated by P (posterior), A (anterior), M (medial) and L (lateral) axes.

Figure 1 shows the OIS responses evoked in SII of two exemplary subjects by contralateral, ipsilateral, and bilateral central pad stimulation. Visual inspection of the images for the three stimulus conditions in each subject shows that: (1) the optical response to contralateral stimulation occurs in a region more anterior in SII than does the response to ipsilateral stimulation; (2) the SII optical response to bilateral stimulation occupies both the anterior and posterior regions that responded to independent stimulation of the contralateral and ipsilateral central pads; and (3) the optical response to ipsilateral stimulation does not evoke a large absorbance change in SI.

**Figure 2:**
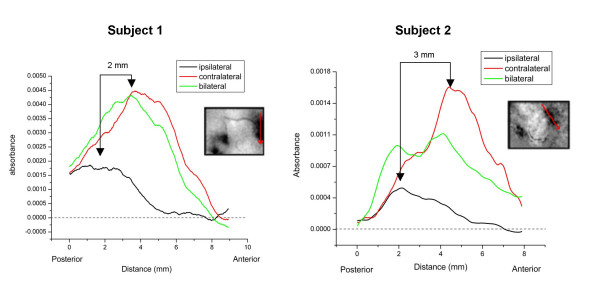
Spatial distribution of response along anterior-posterior axis of SII. Graphs obtained from OIS data in the anterior and posterior SII cortical regions evoked by flutter stimulus on the central pad of the two subjects shown in Figure 1. Orientation of segmentation indicated by figurines. **Left Panel: **Note that the response evoked by the bilateral stimulus is slightly smaller than the response evoked by the contralateral stimulus in the anterior region but larger than the response evoked by the contralateral stimulus in the posterior region. **Right Panel: **Note that in this case, the absorbance values evoked by the bilateral stimulus is significantly smaller than the response evoked by the contralateral stimulus in anterior SII, but approximates a summation of the ipsilateral and contralateral responses in posterior SII. The range of the responses to bilateral stimulation in anterior SII is typified by these 2 subjects – though the differences between the bilateral and contralateral response ranged from small (as in subject 1) to large (as in Subject 2) the bilateral response under the conditions studied were weaker than the contralateral response for all subjects (n = 6).

To more accurately characterize the spatial disparity between SII loci activated by contralateral vs. ipsilateral stimulation, the absorbance values were obtained and plotted along the posterior-anterior axis of SII. Figure 2 shows an absorbance vs. distance plot for each of the 2 subjects whose images are shown in Figure 1. Note that the distance between the peak absorbance values obtained under the ipsilateral and contralateral conditions is approximately 2 mm for Subject 1 (plots on left) and approximately 3 mm for Subject 2 (plots on right). The average across-subject (n = 6) distance between the peaks of SII activation evoked by contralateral and ipsilateral stimulation is 2.4 +/- 0.46 mm [2]. Interestingly, for both subjects, in the posterior region of SII, the magnitude of the response to bilateral stimulation exceeds that of the response to either contralateral or ipsilateral stimulation, but, in the anterior region of SII, the magnitude of response to contralateral stimulation is greater than that of the response to bilateral stimulation. The center of the distribution of the peaks of absorbance evoked by contralateral and ipsilateral stimulation, such as those shown in Figure 2, were used to define the anterior and posterior regions of SII in subsequent analyses (e.g., the peaks were used as the center point of the sampled regions of interest).

**Figure 3:**
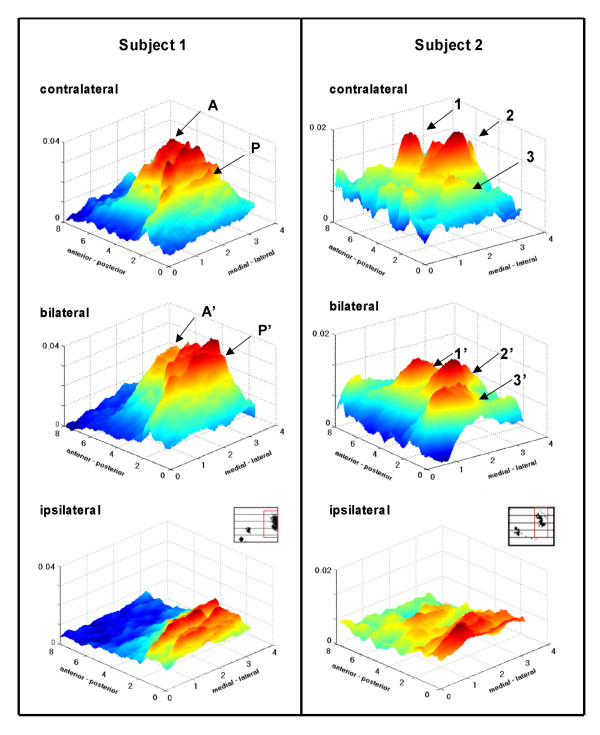
Comparison of contralateral vs. bilateral response in SII. Data displayed is a subset of the data displayed in Figure 1. Region of interest is indicated in figurines. X and Z axes are distance, indicated in mm (along either the anterior-posterior axis or medial-lateral axis). Y axis is absorbance. In Subject 1, note the increase in absorbance in the posterior region in the bilateral response, as compared to the contralateral response (P'>P). Also note the slight decrease in absorbance in the anterior region in the bilateral response relative to the contralateral response (A'<A). In Subject 2, note that in the modules identified in anterior SII (1 & 2), the absorbance value at loci 1 & 2 are greater than their counterparts in the bilateral response (1' and 2'). Module 3, on the other hand, is located in posterior SII (the region of maximal ipsilateral activation), and the absorbance values at 3' are much greater than the values at 3.

A more comprehensive view of the SII response to the contralateral and bilateral stimulus conditions can be better appreciated with a multi-dimensional surface plot of the data. Figure 3 compares the stimulus evoked response of SII in the two subjects to contralateral and bilateral stimulation, and from these plots, it is quite apparent that a large area (in the medial-lateral dimension) of the anterior region of SII is suppressed in the bilateral stimulus condition, relative to the response evoked by the contralateral stimulus. The surface plots of Figure 3 enable direct comparison of specific modules that exhibit a particular profile in one condition (e.g. the contralateral response of Subject 2 – see locations marked 1, 2 and 3) with that of another condition (compare with modules marked 1', 2' and 3' for the bilateral response). In this comparison, the absorbance values at loci 1 and 2 (in anterior SII) are clearly larger than those at locus 3 (in posterior SII) in the contralateral response, and the absorbance values at locations 1' and 2' (from the bilateral response) are much smaller than those at loci 1 and 2 of the contralateral response. Additionally, the response at locus 3 (posterior SII) in the contralateral response is very weak, but is larger in the response to the bilateral stimulus. Thus, the increase in activity observed at locus 3' (i.e., 3'>>3), in the posterior region of SII, evoked by bilateral stimulation parallels a decrease in activity at loci 1' and 2', in the anterior region of SII. In the case of Subject 1, the decrease in activity in anterior SII is not as pronounced as that seen in Subject 2 (also note difference in Figure 2), although the concurrent increase in posterior SII activity (compare module P with P') with decreased anterior SII activity (compare module A with A') is consistent with the shift in activation along the posterior-anterior axis observed in Subject 2. Responses evoked by ipsilateral stimulation are also displayed in Figure 3. Note that in both Subjects 1 and 2, absorbance values in the posterior region are much greater than those in the anterior region of SII.

**Figure 4:**
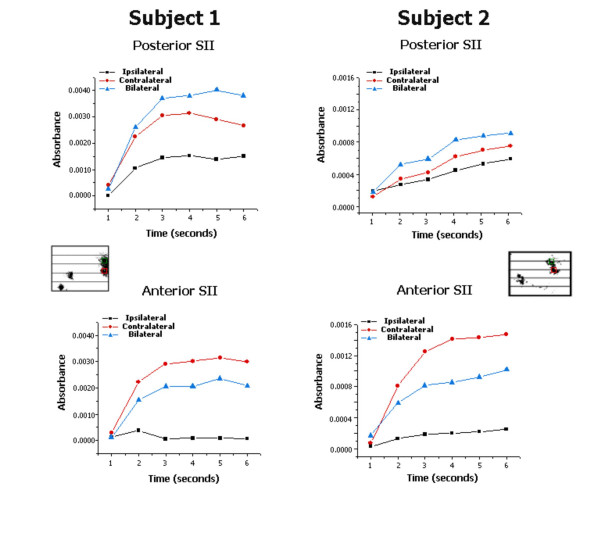
Graphs obtained from OIS data in the anterior and posterior SII cortical regions evoked by flutter stimulus on the central pad of 2 subjects. Figurines indicate regions of interest. **(A) Top Panels: **Time course of absorbance values from the posterior SII region obtained with ipsilateral, contralateral, and bilateral stimulus. Note that in this case, the absorbance evoked by the bilateral stimulus is larger than the response evoked by either the ipsilateral or the contralateral stimulus. **(B) Bottom Panels: **Time course of absorbance values from the anterior SII region obtained from data sampled during ipsilateral, contralateral, and bilateral stimulation. Note that the response evoked by the bilateral stimulus is smaller than the response evoked by the contralateral stimulus. Maximal differentiation of the time course of the response to the different stimulus conditions appears to occur between 1 and 3 seconds.

To directly compare the time course of the response of the anterior and posterior regions of SII to the three stimulus conditions, we determined the time course of the absorbance changes in each region under each stimulus condition (Figure 4). The plots in Figure 4 show that in each of these 2 subjects and under each stimulus condition, the magnitude of the absorbance change evoked in either the posterior or anterior region of SII by ipsilateral stimulation was less than that evoked in the same region by contralateral or bilateral stimulation. Moreover, in both subjects, the magnitude of the response of the posterior region to bilateral stimulation was greater than that evoked by contralateral stimulation, whereas in the anterior region of SII, the magnitude of the response evoked by bilateral stimulation is either less than or approximately equal to the response evoked by contralateral stimulation.

**Figure 5:**
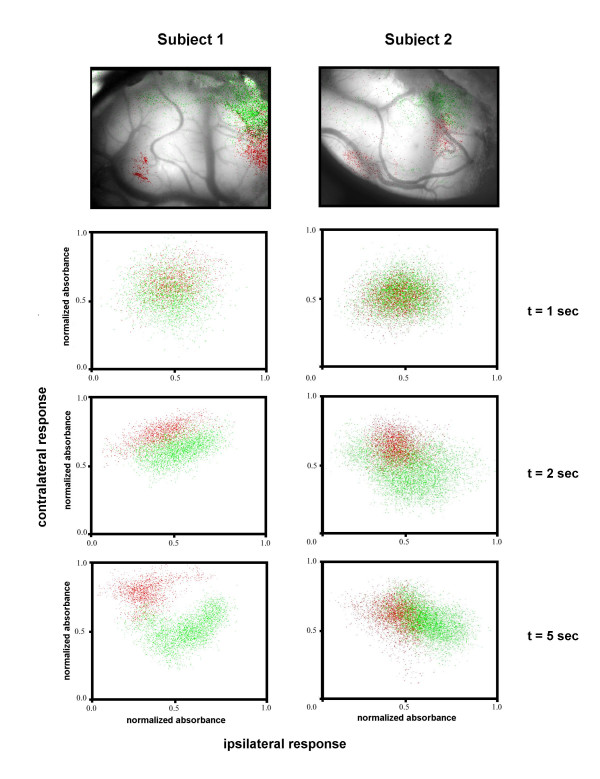
Cluster plots of ipsilateral vs. contralateral response of 2 subjects. For each cluster plot, values of individual pixels are plotted as a function of the response measured at that pixel to the ipsilateral stimulus (horizontal axis) vs. the response measured at that locus evoked by the contralateral stimulus (vertical axis). Colors depict the pixels that maximally responded to ipsilateral (green) and contralateral (red) stimulation. Cluster separation follows the same trend as the time course shown in Figure 4. After 5 seconds, the activity of the responding population has diverged into two distinct clusters. Reference images at top are at same orientation as the reference images in Figure 1.

Cluster plots were used to directly compare the response of SII to the different conditions of ipsilateral and contralateral stimulation. In each plot in Figure 5, the absorbance value obtained at each pixel to the 2 different stimulus conditions is plotted against each other – i.e., the x-axis is the absorbance value evoked by the contralateral stimulus and the y-axis is the absorbance value evoked by the ipsilateral stimulus. The clusters reveal a distinct differentiation in the population of SII neurons to the different stimulus conditions. Additionally, it appears that this could be a time dependent process, as there is little difference in the behavior of the pixels localized to the SII region in the early stages of the response (t = 1 sec), there is some grouping after 2 seconds, and there are two distinct clusters formed after several seconds (t = 5 sec). It should be emphasized that this type of graphic does not necessarily reflect spatial differences in the responses of two different stimulus conditions, but rather, it emphasizes whether or not different members of a set respond differently to different stimulus conditions. Thus, the information demonstrated by the cluster plots in Figure 5 can be summarized by stating that with an increase in stimulus duration (from 1 to 5 seconds), there is an increase in the segregation of the behavior of the different groups of pixels whose values are more predominantly affected by a contralateral vs. an ipsilateral stimulus.

**Figure 6:**
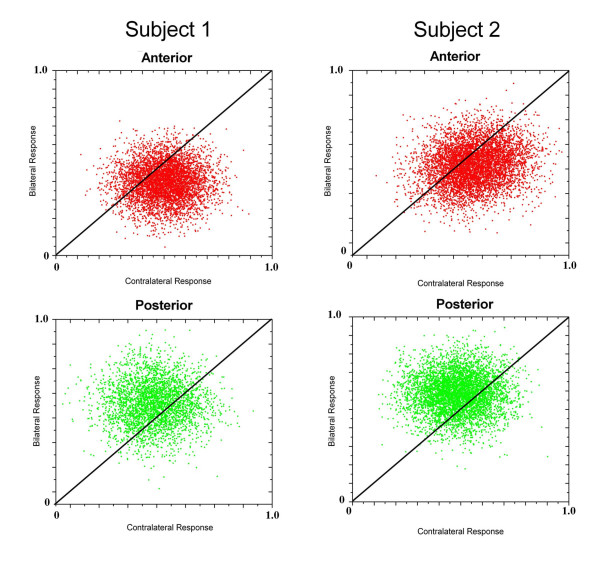
Cluster plots of contralateral vs. bilateral response of 2 subjects. For each cluster plot, values of individual pixels are plotted as a function of the absorbance measured at that pixel to the contralateral stimulus (horizontal axis) vs. the response measured at the same locus (or pixel) evoked by the bilateral stimulus (vertical axis). Reference line, plotted at a slope of 1, indicates where pixels with equal values for both conditions lie. Note that in the anterior region, the majority of the pixels are below the reference line (response to bilateral stimulus was weaker than the response to the contralateral stimulus) and that in the posterior region, the majority of the pixels are plotted above the reference line (response to bilateral stimulus is greater than the response to the contralateral stimulus). Plots are normalized (minimum absorbance value scaled to 0; maximum scaled to 1).

The contralateral and bilateral stimulus conditions can also be compared using cluster analysis. However, because the difference between the contralateral and ipsilateral responses is more robust than the difference between the contralateral and bilateral responses, Figure 6 displays the results of cluster analysis of the anterior and posterior regions of SII independently. Independent analysis of the two regions allows for better resolution of shifts in the behavior of activity within particular regions. For each subject, the peak response was identified in both the anterior and posterior regions. Cluster plots were obtained by plotting the response (contralateral along the x-axis, bilateral along the y-axis) at each location within a 1 × 1 mm^2 ^boxel surrounding the peak. In anterior SII, the majority of the responses to contralateral stimulation are stronger than the responses to bilateral stimulation – hence, the majority of the points plotted fall below the reference line (which has a slope of 1). In the posterior region, the majority of the points plotted are above the reference line – indicating that the response to the bilateral stimulus was greater than the response to the contralateral stimulus.

**Figure 7:**
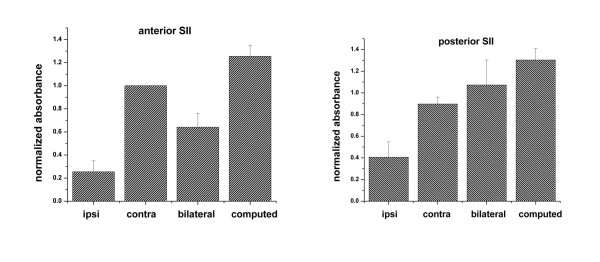
Averaged normalized absorbance values (n = 6) obtained at 3 seconds after stimulus onset with respect to the contralateral stimulus in the anterior and posterior SII region. Computed response is the summation of the ipsilateral and contralateral responses. **Left Panel: **The contralateral stimulus in the anterior SII region evoked the greatest cortical response, while the ipsilateral stimulus was significantly lower. The bilateral response, however, evoked a cortical response that fell between the ipsilateral and the contralateral evoked responses. Note that the bilateral response is lower than the computed response. **Right Panel: **The ipsilateral response in the posterior SII region is greater than the ipsilateral response in the anterior SII region. The difference between the ipsilateral and contralateral responses is not as great in the posterior SII region as it was observed in the anterior SII region. Note that the bilateral response is larger than both the ipsilateral and contralateral responses, but still remains less than the computed value.

Thus far, the results suggest that the anterior and posterior regions of SII are differentially activated by contralateral, ipsilateral and bilateral stimulation. To determine the across-subject consistency of these findings, the average absorbance values evoked by the 3 different stimulus conditions were determined for all 6 of the subjects (Figure 7). Clearly, in the anterior region of SII, the contralateral stimulus condition evoked the largest magnitude of response, and therefore, the values of the absorbance increase obtained in this and the posterior region under each stimulus condition were normalized to this absorbance value (thus, standard error for the contralateral/anterior region condition = 0). Whereas in the anterior region of SII, the response evoked by bilateral stimulation is approximately 35% less than that evoked by contralateral stimulation, in the posterior region of SII, the bilateral stimulus evoked a response approximately 25% larger than that evoked by the contralateral stimulus. Analysis of variance showed that, at a 95% confidence interval, the average bilateral:contralateral response ratio was between 0.42 and 0.83 in the anterior region of SII. In the posterior region of SII, the same analysis showed the bilateral:contralateral response ratio to be between 0.97 and 1.43 at a 95% confidence interval. Although the response of the posterior region to the bilateral stimulus is larger than that evoked by the contralateral stimulus, it is less than that predicted by summation of the responses to the ipsilateral and contralateral stimuli (computed values, Figure 7). Furthermore, while the response of neither the anterior or posterior regions of SII to bilateral stimulation approximate a linear summation of the responses to independent stimulation of each central pad, the approximation of the bilateral response by summation of responses evoked by independent stimuli is much closer to being accurate in the posterior region of SII.

## Discussion

The findings of this study demonstrated clearly that the anterior and posterior regions of SII process bilateral inputs very differently. At the locus of the maximal OIS response evoked in the posterior region by an ipsilateral stimulus, bilateral stimulation evoked a response that was, on average, about 25% larger than that evoked from the contralateral stimulus site. Conversely, at the locus of the maximal OIS response evoked by contralateral stimulation in the anterior region, bilateral stimulation evoked a response that was, on average, 35% lower than the activity evoked by a contralateral stimulus. This discrepancy between the optical responses of the anterior and posterior regions could be related to neurophysiological observations reported in earlier studies. For example, Carreras and Andersson [3] found that for a sizable fraction of the cat SII neurons in their study, ipsilateral mechanical skin stimulation inhibited the response to contralateral stimulation, whereas in contrast, Picard et al. [4] found in their study of neurons in the distal forelimb regions of cat SII that simultaneous delivery of contralateral and ipsilateral mechanical skin stimuli led to strong facilitation of SII neuron response. In the study of Picard et al. [4], the responses of cells to bilateral stimulation were found to exceed the stronger of the responses to unilateral stimulation by, on average, 230%. Their study was limited, however, to the very low numbers of SII neurons that had bilateral RFs on the distal limbs. Burton, et al. [5], similar to Carreras and Andersson [3], reported that SII cells with bilateral receptive fields (monkey) exhibited a reduction in mean firing rate of 30% when the contralateral stimulus was preceded by an ipsilateral stimulus. Finally, other workers have found that callosally-transmitted inputs tend to have excitatory effects on SII neurons that have bilateral RFs, and exert inhibitory effects on SII neurons that have exclusively contralateral RFs [6-8]. Simoes et al. [9] showed significant suppression of the MEG SII response in humans, with simultaneous inputs delivered to the same skin sites, and Hoechstetter et al. [10] described "interactions" in SII cortex (a response that was not the summation of the ipsilateral and contralateral response) to simultaneous bilateral stimuli. Definitive establishment of the relationship between stimulus-evoked SII neuroelectrical and OIS activation, however, must await the performance of combined imaging and neurophysiological investigations which utilize both methodologies in the same subjects and under the same stimulus conditions.

The main, although not the only, route for ipsilateral input to SII is through the corpus callosum, from cells located in SI and SII of the opposite cerebral hemisphere [4,7,8,11]. Even those regions in SII that represent most distal parts of the limbs receive significant numbers of connections from the homologous zones of the contralateral SI and SII [12-15]. Graziosi [16] showed that separate populations of cells in SI provide callosal projections to SI and SII in the opposite hemisphere and ipsilateral projections to SII. Some separation within SII of the responses to ipsilateral and contralateral stimulation was also shown by Friedman et al. [17] and Juliano et al. [18].

The neurons in the distal limb regions of SII do receive substantial callosal connections, but these neurons have been reported to lack ipsilateral RFs [1], indicating that callosal inputs are not strong enough to generate action potentials (at least under the conditions used in RF mapping studies). This suggests that SII neurons do not use their sensory inputs from the ipsilateral side of the body to construct functional properties dependent on bilateral inputs; in other words, to extract information about higher-order properties of bi-manually contacted objects from coordinated patterns of sensory stimulation of the two hands. Instead, it could be postulated that neurons in the distal limb regions of SII use their ipsilateral peripheral inputs to modulate the responses to contralateral peripheral stimulation. On the other hand, Bennett et al. [19] found that bilateral convergence on SII neurons varies markedly with the different classes of tactile neurons, and modulation of the SII response by ipsilateral inputs may vary from one cortical area to another with different stimulus modalities.

A number of interactions between stimuli applied to both hands have been demonstrated in human psychophysical studies. Gilson [20] found that the threshold for detection of vibrotactile stimuli applied to a fingertip is elevated by parallel stimulation of the other hand's fingers. In addition, Gescheider and Verrillo [21] reported that the magnitude of vibrotactile sensation, elicited by brief 25 or 300 Hz stimuli applied to thenar eminence, was decreased by stimuli applied simultaneously to the opposite hand, but was enhanced when the contralateral stimulus was applied 150 msec prior to the test stimuli. Essick and Whitsel [22] reported that the perception of the direction of motion of brushing stimuli on the skin is enhanced by the presence of a simultaneous contralateral brushing stimulus when the two stimuli move in the same direction, but is weakened when the contralateral stimulus moves in a direction opposite to that on the other arm. While the above described reports provide possible perceptual correlates for bilateral interactions that might occur in SII, such as those identified in the present study, it will remain uncertain until anterior or posterior SII cortical activity is studied under conditions that permit direct correlations of perceptual performance and cortical activity under precisely controlled conditions of contralateral vs. bilateral skin stimulation.

A recent report [23] demonstrated 3 separate functional cortical fields along the anterior-posterior axis in the macaque. These functional fields were defined based on differential neural responses from three distinct cortical fields, and their report was unique in that it described cortical areas within SII based on functional properties of cortical areas. In this report, we demonstrate at least two functional subdivisions within SII in the cat based on functional properties as well. However, the modes of stimulation used to distinguish the functional differences along the anterior-posterior axis of SII were very different in this study (contralateral/ipsilateral/bilateral vs. proprioceptive/cutaneous inputs in the Fitzgerald study), and subsequent investigations using other stimulus modalities could reveal that SII of the cat is organized in a very similar fashion to SII of primates. The multiple fields found in SII, based on functional differences, could be, as suggested by Fitzgerald, et al. [23], indicative of the existence of a number of distributed processing streams. The significance of the presented work is that the response of these different cortical areas, which could represent information from so-called separate information streams, changes in a manner dependent upon the activity of neighboring cortical areas. Distinction of cortical areas within SII, identified by functional characteristics, demonstrates the nonlinearity of the integration of information from different sources (or information streams).

One question that the results suggest is whether or not SII can be segregated by laterality preference, in a manner similar to that observed in other sensory systems. Laterality has been demonstrated in the primary sensory cortex of both the visual system and the auditory system of both primates and cats, and the data in this report strongly suggest that there are cortical areas within SII that exhibit preference to ipsilateral or contralateral inputs. In terms of processing information from simultaneous contralateral and ipsilateral stimuli, there could be further similarities between the somatosensory, auditory and visual systems that have yet to be described. Future investigations will aim to further clarify the role of SII in integration of information from inputs across the body midline.

## Conclusions

The responses evoked by contralateral and ipsilateral flutter stimulation of the central pad of the cat forepaw define functional subdivisions in SII: the two modes of stimulation maximally activate cortical regions that are anterior and posterior to one another, respectively. Bilateral stimulation, or providing simultaneous contralateral and ipsilateral stimulation, reveals, additionally, that the two adjacent cortical areas process bilateral inputs differently. In the posterior region, where ipsilateral stimulation evokes a maximal response, bilateral stimuli evoke a response that is greater than the response evoked by either the individual ipsilateral or contralateral response. In the anterior region of SII, where the contralateral stimulus evokes a maximal response, bilateral stimuli evoke responses that are smaller in magnitude than the responses evoked by the contralateral stimulus.

## Methods

### Subjects & preparation

Adult cats (males and females; n = 6) were subjects. All surgical procedures were carried out under deep general anesthesia (1 – 4% halothane in a 50/50 mixture of oxygen and nitrous oxide). After induction of general anesthesia the trachea was intubated with a soft tube and a polyethylene cannula was inserted in the femoral vein to allow administration of drugs and fluids (5% dextrose and 0.9% NaCl). For each subject, a 1.5 cm diameter opening was made in the skull overlying somatosensory cortex, a chamber was mounted to the skull over the opening with dental acrylic, and the dura overlying anterior parietal cortex was incised and removed. Following the completion of the surgical procedures all wound margins were infiltrated with long-lasting local anesthetic, the skin and muscle incisions were closed with sutures, and each surgical site outside the recording chamber was covered with a bandage held in place by adhesive tape.

Subjects were immobilized with Norcuron and ventilated with a gas mixture (a 50/50 mix of oxygen and nitrous oxide; supplemented with 0.1 – 1.0% halothane when necessary) delivered via a positive pressure respirator 1–3 hours prior to the data acquisition phase of the OIS imaging experiments. Respirator rate and volume were adjusted to maintain end-tidal CO_2 _between 3.0 – 4.0%; EEG and autonomic signs (slow wave content; heart rate, etc.) were monitored and titrated (by adjustments in the anesthetic gas mixture) to maintain levels consistent with light general anesthesia. Rectal temperature was maintained (using a heating pad) at 37.5°C.

Euthanasia was achieved by intravenous injection of pentobarbital (45 mg/kg) and by intracardial perfusion with saline followed by fixative (10% formalin). Following perfusion fiducial marks were placed to guide removal, blocking, and subsequent histological sectioning of the cortical region studied. All procedures were reviewed and approved in advance by an institutional committee and are in full compliance with current NIH policy on animal welfare.

### Stimuli and stimulus protocols

Results were obtained during stimulation of the contralateral central pad of the forepaw and/or the ipsilateral central pad of the forepaw. The stimuli always consisted of sinusoidal vertical skin displacements (25 Hz, 400 microns, stimulus duration 5 – 20 sec, inter-stimulus interval 60 sec) and were applied using a servocontrolled transducer (Cantek Enterprises, Canonsburg, PA) that is capable of delivering sinusoidal stimuli in the range of 1–250 Hz at amplitudes in the range of 0–1000 microns. The stimuli were delivered independently to the ipsilateral and contralateral skin sites, and also were applied simultaneously to both sites (bilateral stimulation). The stimulus probes were positioned 500 microns beyond the point at which skin contact was detected (via force transducer on the Cantek). The bilateral stimulus protocols reported in this paper were synchronized to start and stop at the same time. The contralateral, ipsilateral and bilateral stimuli were interleaved on a trial-by-trial basis. This approach was used to control for temporal changes in cortical "state" unrelated to stimulus conditions which, if unrecognized, might obscure or modify any differences between the optical responses evoked by the contralateral, ipsilateral and bilateral stimulus conditions.

### OIS imaging

Near-infrared (IR; 833 nm) OIS imaging was carried out using an oil-filled chamber capped with an optical window [24]. Images of the exposed cortical surface were acquired 200 msec before stimulus onset ("reference" or "prestimulus" images) and continuously thereafter ("poststimulus" images; at a resolution of one image every 0.5 to 1.5 sec) for 15–20 sec following stimulus onset. Exposure time was 200 msec. Absorbance images were generated by subtracting each prestimulus (reference) image from its corresponding poststimulus image and subsequently dividing by the reference image. Averaged absorbance images typically show regions of both increased absorption of IR light and decreased absorption of light (to a depth of approximately 1400 microns) which have been shown to be accompanied by increases and decreases in neuronal activation, respectively [24-29].

### Histological procedures/identification of cytoarchitectural boundaries

At the conclusion of the experiment, the imaged cortical region was removed immediately following intracardial perfusion with saline and fixative. The region then was blocked, postfixed, cryoprotected, frozen, sectioned serially at 30 μm, and the sections stained with cresyl fast violet. The boundaries between adjacent cytoarchitectonic areas were identified by scanning individual sagittal sections separated by no more than 300 μm and were plotted at high resolution using a microscope with a drawing tube attachment. The resulting plots then were used to reconstruct a two-dimensional surface map of the cytoarchitectonic boundaries within the region studied with optical and neurophysiological recording methods. The locations of microelectrode tracks and electrolytic lesions evident in the histological sections were projected radially to the pial surface and transferred to the map of cytoarchitectonic boundaries reconstructed from the same sections. As the final step, the cytoarchitectonic boundaries (along with the locations of microelectrode tracks and lesions whenever present) identified in each brain were mapped onto the images of the stimulus-evoked intrinsic signal obtained from the same subject, using fiducial points (made by postmortem applications of india ink or needle stabs) as well as morphological landmarks (e.g., blood vessels and sulci evident both in the optical images and in histological sections). Locations of cytoarchitectonic boundaries were identified using established criteria [30-32].

## Abbreviations

A-P = anterior-posterior

RA = rapidly adapting

SA = slowly adapting

PC = Pacinian

RF = receptive field

OIS = optical intrinsic signal

IR = near infrared

EEG = electro-encephalogram

MEG = magneto-encephalogram

## Authors' contributions

BW and OF participated in the design of the experiments, the data collection, and drafting of the manuscript. SS, JC, and VT made significant contributions to the data collection and the analysis of the data. MT played a major role in all aspects of the development of the manuscript.
